# A Harsh Environment-Oriented Wireless Passive Temperature Sensor Realized by LTCC Technology

**DOI:** 10.3390/s140304154

**Published:** 2014-03-03

**Authors:** Qiulin Tan, Tao Luo, Jijun Xiong, Hao Kang, Xiaxia Ji, Yang Zhang, Mingliang Yang, Xiaolong Wang, Chenyang Xue, Jun Liu, Wendong Zhang

**Affiliations:** 1 Key Laboratory of Instrumentation Science & Dynamic Measurement, Ministry of Education, North University of China, Tai Yuan 030051, China; tanqiulin.99@163.com (Q.T.); 18734135497@163.com (H.K); ji418xiaxia@163.com (X.J.); zhang418yang@163.com (Y.Z.); yang418mingliang@163.com (M.Y.); wangdakeliou@126.com (X.W.); xuechenyang@nuc.edu.cn (C.X.); Liuj@nuc.edu.cn (J.L.); wdzhang@nuc.edu.cn (W.Z.); 2 Science and Technology on Electronic Test & Measurement Laboratory, North University of China, Tai Yuan 030051, China

**Keywords:** temperature sensor, LTCC, harsh environments, ferroelectric dielectric material, wireless passive

## Abstract

To meet measurement needs in harsh environments, such as high temperature and rotating applications, a wireless passive Low Temperature Co-fired Ceramics (LTCC) temperature sensor based on ferroelectric dielectric material is presented in this paper. As a LC circuit which consists of electrically connected temperature sensitive capacitor and invariable planar spiral inductor, the sensor has its resonant frequency shift with the variation in temperature. Within near-filed coupling distance, the variation in resonant frequency of the sensor can be detected contactlessly by extracting the impedance parameters of an external antenna. Ferroelectric ceramic, which has temperature sensitive permittivity, is used as the dielectric. The fabrication process of the sensor, which differs from conventional LTCC technology, is described in detail. The sensor is tested three times from room temperature to 700 °C, and considerable repeatability and sensitivity are shown, thus the feasibility of high performance wireless passive temperature sensor realized by LTCC technology is demonstrated.

## 1. Introduction

As a fundamental physical quantity, temperature is the most universal and important processing parameter in the production process. Temperature measurement is indispensable in many aspects, such as heat treating, aluminum electrolysis [1], metallurgy, aviation and aerospace, *etc.* However, accurate temperature measurement is still a challenge in harsh environments, such as high temperature, hermetic space and rotating components.

Various temperature sensors are investigated to realize accurate temperature measurement in these harsh environments. Among those sensors, surface acoustic wave (SAW) sensors and wireless passive LC sensors are considered as suitable choices due to their characteristics of no batteries and wireless signal readout. The SAW temperature sensor was investigated in many previous publications, such as a SAW-RFID-enabled temperature sensor proposed by a group at Shanghai Jiao Tong University, which performs well within 40 °C [2]. A temperature monitoring method based on a SAW temperature sensor which extends the measurement range to 250 °C is proposed in [3]. Although a far readout distance can be obtained with SAW temperature sensors, the signal can be easily influenced by the velocity of sound, which is strongly dependent on not only temperature, but also environmental, geometric and material properties along the path. For their small size and stable characterization, wireless passive LC sensors are particularly suitable for temperature measurement in some harsh environments, such as hermetic spaces and rotating components. A group from the University of Puerto Rico proposed a wireless passive temperature sensor which takes high-k ferroelectric ceramic as capacitor dielectric, and good sensitivity under 235 °C is shown [4]. A contactless thermistor-based measurement of temperature was investigated by a group at Zhejiang University [5]. A passive wireless temperature sensor based on a temperature sensitive oscillator sensor circuit is presented in [6], which can work well only at temperatures below 200 °C. Andò presented a contactless temperature sensor based on a MEMS temperature sensitive structure which can work up to 500 °C [7]. A wireless passive temperature sensor based on a MEMS structure was also investigated by Marioli [8]. A paper from the 2006 International MEMS Conference proposed a passive telemetry LC absolute pressure and temperature sensor for a Tire Pressure Monitoring System (TPMS), which is designed to work under 100 °C based on temperature dependent resistivity [9]. Unger proposed a passive wireless temperature sensor based on LTCC technology for the first time [10] and investigated it in depth in his later paper [11]. A temperature sensor based on temperature sensitive LTCC ceramic was also investigated by Radosavljevic and a sensitivity of 1 KHz/°C within a measurement range of 200 °C was obtained [12]. For harsh environment-oriented applications, the existing wireless passive temperature sensors, which have measurement ranges below 500 °C with complex fabrication technology or low sensitivity, should be investigated and optimized further.

Based on the basic principle of a wireless passive LC sensor, this paper proposes a sensor which takes a high Curie ferroelectric dielectric material as temperature sensitive dielectric and fabricated by LTCC technology. It has advantages of low cost, simple fabrication technology and self-assembly. The chemical and physical stability of LTCC substrate makes it perform well in high temperature and corrosive environments. Temperature characteristics of the sensor are tested from room temperature to 700 °C. Measurement results show the considerable sensitivity and repeatability of the sensor.

## 2. Sensor Principle

**Figure 1.**
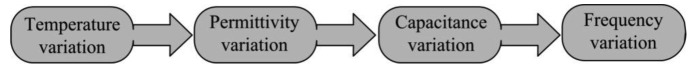
System block diagram of the proposed sensor.

**Figure 2.**
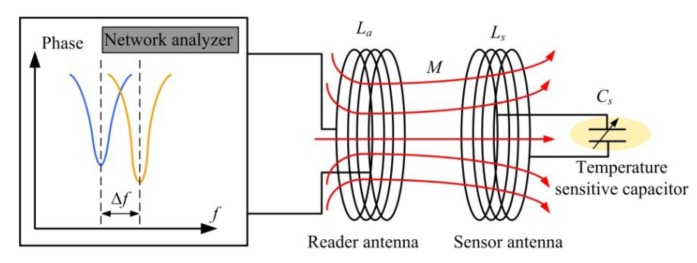
Telemetric inductive model of the sensor.

The proposed sensor is equivalent to a LC circuit, which has a capacitance sensitive to temperature. As shown in Figure 1, ambient temperature variation causes a variation in the permittivity *ε_r_* of the sensitive material, and then a capacitance change, which results in a change of the resonance frequency of the sensor. Based on the principle of resonant coupling, as shown in Figure 2, the change in resonance frequency can be detected by external coil antenna, which is magnetically coupled to the sensor. A sweep signal which covers the variation range of resonance frequency of the sensor is inputted into the antenna port, when the transmitting frequency equal to the resonance frequency of the sensor, resonance oscillation occurs, and the input impedance looking into the antenna change abruptly [13].

By measuring the impedance magnitude and phase, resonance of the sensor can be retrieved. From the circuit analysis, the impedance phase ∠*Z*_1_ can be deduced as [14]:

(1) $$∠Z_{1}=\text{arctan}\left[\frac{\left(1-\Omega^{2})+\frac{\Omega^{2}}{Q^{2}}+k^{2}\Omega^{2}(1-\Omega^{2}\right)}{\frac{k^{2}\Omega^{3}}{Q}}\right]$$

Here, Ω is defined as *f*/*f_0_*, and *f* is the sweep frequency, *f_0_* is the resonance frequency of the sensor which can be written as:

(2) $$f_{0}=\frac{1}{2\pi \sqrt{L_{s}C_{s}}}=\frac{1}{2\pi \sqrt{L_{s}(C_{\text{plate}}+C_{\text{par}})}}$$

where *L_s_* is the series inductance, *C_s_* is the series capacitance, *C_plate_* is the capacitance of parallel capacitor, and *C_par_* is the parasitic capacitance of sensor coil. Compared with *C_plate_*, *C_par_* is quite small, so the resonant frequency of the sensor is mainly influenced by *C_plate_*. The quality factor *Q* can be given by:

(3) $$Q=\frac{2\pi f_{0}L_{s}}{R_{s}}=\frac{1}{R_{s}}\sqrt{\frac{L_{s}}{C_{s}}}$$

where *R_s_* is the series resistance of the sensor coil, *k* in Equation (1) is the coupling coefficient between the read antenna and the sensor coil, it is defined as:

(4) $$k=\frac{M}{\sqrt{L_{a}L_{s}}}$$

Here, *M* is the mutual inductance between the antenna and the sensor coil, *L_a_* is the self-inductance of the reader antenna. By further mathematical deduction, the frequency corresponding to the impedance phase negative peak *f_min_* can be written as [14]:

(5) $$f_{\min}=f_{0}(1+\frac{k^{2}}{4}+\frac{1}{8Q^{2}})$$

For practical applications, *k* is small and *Q* is big enough to make *f_min_* approach *f_0_*, so the variation of resonant frequency *f_0_* can be retrieved by detecting the change of *f_min_*.

## 3. Sensor Design and Fabrication

**Figure 3.**
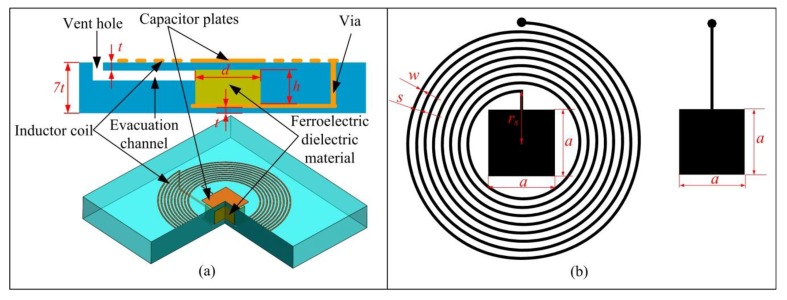
(**a**) Cross-section and 3D diagram of the sensor; (**b**) layout of the sensor.

The cross-section diagram of the sensor is illustrated in Figure 3a. The sensor consists of a planar spiral inductor and a parallel-plate capacitor in which the ferroelectric ceramic is used as dielectric. The planar spiral inductor is electrically connected to the capacitor by vias, which form a passive LC resonator with temperature dependent resonant frequency. The layout of the sensor is shown in Figure 3b and its designed dimension parameters are indicated. The designed values for the dimensions in Figure 3 are listed in Table 1.

The low frequency self-inductance, *L_s_*, of a circular planar spiral inductor can be estimated by:

(6) $$L_{s}=\frac{\mu_{0}n^{2}d_{\text{avg}}}{2}\left[\ln(\frac{2.46}{\varphi_{s}})+0.2\varphi_{s}^{2}\right]$$

where *μ_0_* is the permeability of free space, which is equal to 4*π* × 10^−7^H/m, *d_avg_* is the average diameter of the spiral coil, and it can be derived as *d_avg_ =* 2*r_s_* + *n*(*s + w*), and *φ_s_* is the fill ratio and can be given as *φ_s_* = *n*(*s + w*)/[2*r_s_* + *n*(*s + w*)]. Substitute the dimension values listed in Table 1 into Equation (6), it can be calculated that *L_s_* at room temperature is 1.345 μH.

The temperature sensitive capacitance of parallel capacitor *C_plate_* can be expressed as:

(7) $$C_{\text{plate}}=\frac{{ɛ}_{0}d^{2}}{\frac{t}{{ɛ}_{\text{rc}}(T)}+\frac{h}{{ɛ}_{\text{rd}}(T)}}+\frac{{ɛ}_{0}(a^{2}-d^{2})}{\frac{6t}{{ɛ}_{\text{rc}}(T)}}$$

where *ε_0_* is the dielectric constant of air, which is equal to 8.85 × 10^−12^F/m. *ε_rc_*(T) and *ε_rd_*(T) are the temperature-dependent permittivity of the LTCC ceramic material and ferroelectric dielectric ceramic separately. When the ambient temperature change, there are variations in *ε_rc_*(T) and *ε_rd_*(T), which result in the drift of capacitance *C_plate_*.

**Figure 4.**
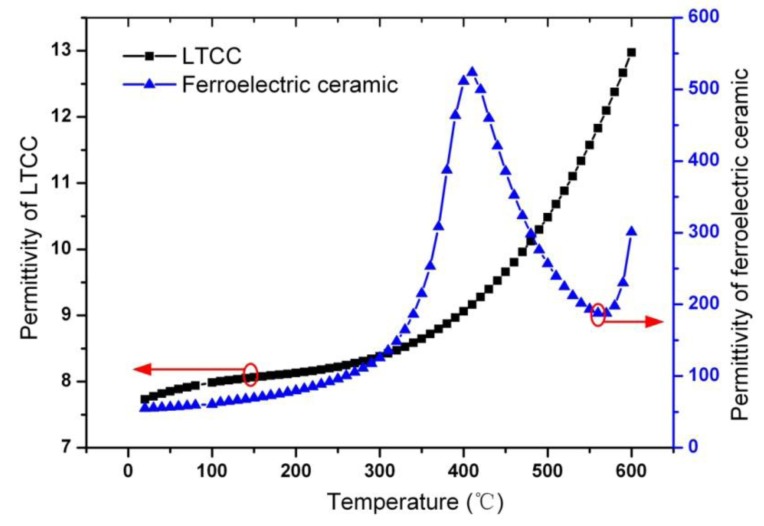
Measurement permittivity of LTCC and ferroelectric ceramic *versus* different temperature.

951 AT LTCC (Dupont, Wilmington, DE, USA) is used as substrate material and PN ceramic from Bao Dding HengSheng Acoustics Electron Apparatus Co., Ltd. (Baoding, China) is used as dielectric material. The PN ceramic is a kind of PbNb_2_O_6_-based ferroelectric ceramic. In order to analyze the temperature characteristics of *C_plate_*, the permittivity of Dupont 951 AT LTCC and ferroelectric ceramic are measured from room temperature to 600 °C at 2MHz at Wuhan Pusite Technologies Co., Ltd. (Wuhan, China) as shown in Figure 4. With temperature increases from room temperature to 600 °C, it can be calculated that *C_plate_* increases from 11.76 pF to 26.49 pF by substituting the measured permittivity of LTCC and ferroelectric ceramic into Equation (7). Although the permittivity of the ferroelectric dielectric ceramic decreases when temperature increases from 409 °C to 570 °C, *C_plate_* increases monotonously with temperature change from room temperature to 700 °C. The reason for the monotone increase of *C_plate_* is due to the rapid increase of permittivity of LTCC ceramic after 400 °C, which compensates for the decrease of permittivity of the ferroelectric dielectric ceramic.

Seven layers of ceramic tapes are used to make up the sensor structure, and the ferroelectric ceramic is embedded in the LTCC substrate which is a difference form conventional LTCC technology. The fabrication process is based on lamination and sintering of screen-printed DuPont 951 AT LTCC and can be broadly divided into eight steps.

The first step is silk screen design and fabrication. CAD software is used to draw the layout of the inductor and capacitor, as illustrated in Figure 3b. The layout is used as mask plate to etch the stainless steel screen, and then sliver paste can be printed onto the LTCC green tape through the corroded area.

**Figure 5.**
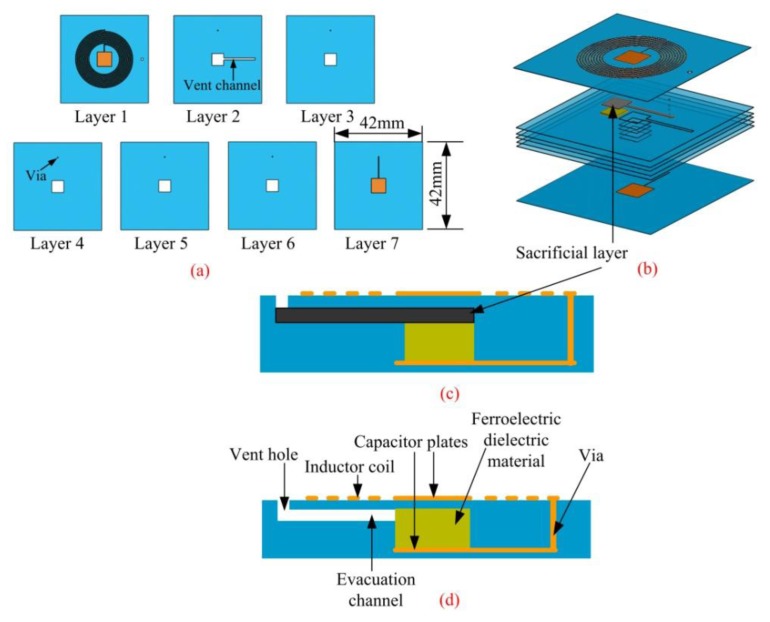
Major fabrication process of the sensor.

The second step is punching. A punching document generated by CAD software, is used by a punching machine to punch the alignment holes, via, cavity and evacuation channel as well as the vent hole, as illustrated in Figure 5a. Alignment holes are used as benchmarks to align the tapes in the process of collating and screen-printing. By filling with conductor paste, the via is used to realize the electrical connection between the different layers.

The third step is via filling. After punching, Dupont 6146D Ag paste is used to fill the vias. First, paste is smeared over the slide of the porefilling machine uniformly. Then, via-screen is put onto the layer of paste and the green tape is put on the via-screen after alignment. At last, this adjusted system is pushed into the porefilling machine, and the top of the bulk additive sucked to implement the via filling. The result of via filling is illustrated in Figure 5a.

The fourth step is conductor printing. Dupont 6142D Ag paste and the silk-screen with resolution of 325 meshes are used for printing the inductor coil and capacitor electrodes. First, the silk-screen is mounted on the screen machine. Then, make the green tape aligned with the screen. After alignment, as illustrated in Figure 5a, the circuitous pattern can be printed on the ceramic tape by precisely controlling the force, velocity and angle of the scraper. The parameter setup of the scraper is quite important for the quality of the printing, for example, if excessive velocity is used, leaky printing or ambiguity of the pattern will occur.

The fifth step is collating. The oven-dried green tapes are stacked by the collating machine according to the designed sequence. Due to the need of filling ferroelectric dielectric material and sacrificial layer [15], the bottom six layers should be collated first, and then the ferroelectric dielectric and sacrificial layers are filled into the cavity sequentially. Finally, the last green tape layer is collated on the top. ESL49000 carbon diagram is used as sacrificial layer to support the upper capacitor plates to avoid the occurrence of crack in the process of lamination, as illustrated in Figure 5b.

The sixth step is lamination. The embryonic plant of the multilayer substrate should be packaged under vacuum to avoid any contact with water. Then, the packaged substrate is put into the laminating machine, in which a pressure of 15 MPa, temperature of 70 °C and time of 20 min are used to process the lamination [16]. The pressure in the process of lamination is isotropic, which guarantees the structural stability of the multilayer substrate, as shown in Figure 5c.

The seventh step is pre-cutting. The laminated multilayer substrate is put into a dry furnace and it is preheated under 70 °C. After 10 min, the substrate is cut into a single sensor sample by a heat-cutting machine. In the process of pre-cutting, the blade and platform should be warmed up to guarantee the smooth and verticality of the cutting face. The temperature of the blade and platform should also be controlled precisely.

**Figure 6.**
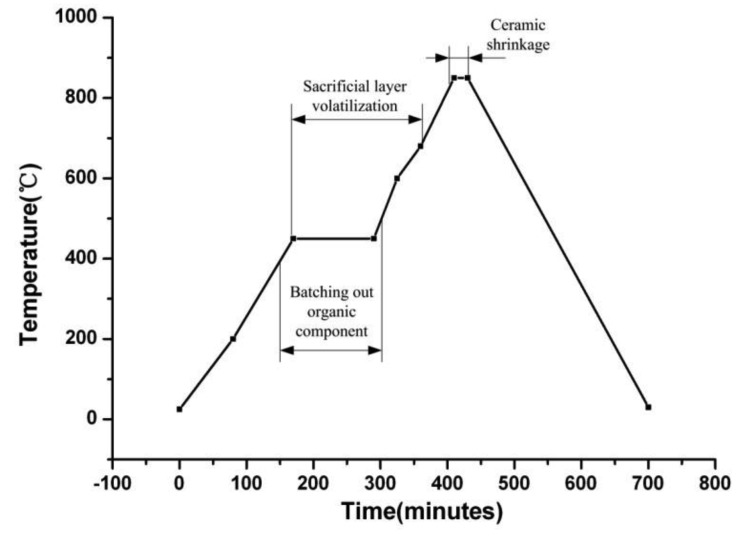
Sintering curve of the sensor.

**Figure 7.**
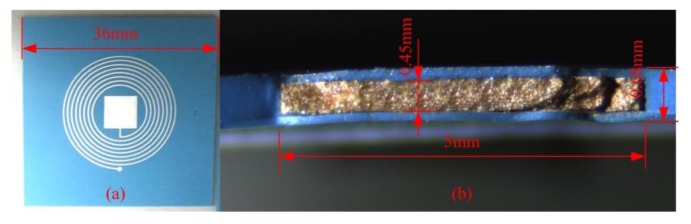
(**a**) Sensor sample and (**b**) its cross-section photograph.

The eighth and final step is co-firing. The sensor samples are put into the sintering furnace and then sintered according to the sintering curve illustrated in Figure 6. After sintering the samples at the peak temperature of 850 °C [17], the former multilayer substrate becomes a rigid integration. Among the process of sintering, carbon diaphragm which is used as sacrificial layer volatilize completely. Also, the dimension of the sensor shrinks, as illustrated in Figure 5d. The sensor sample after sintering is illustrated in Figure 7.

## 4. Measurement Results

**Figure 8.**
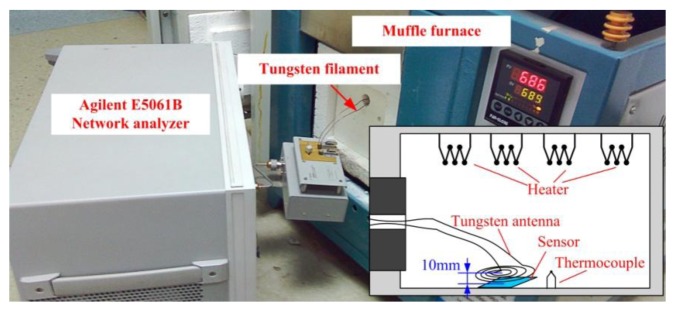
Temperature characteristics test system of the sensor.

The sensor sample after sintering is illustrated in Figure 7. The resonant frequency of the sensor is tested from room temperature to 700 °C by using the high temperature measurement system which consists of a muffle furnace and an E5061B Network Analyzer, as shown in Figure 8. The sensor is placed below the reader antenna which is coiled by a tungsten filament due to its better stability at high temperature. The tungsten coil has a maximum radius of 35 mm, and the distance between the LTCC sensor and the antenna is about 10 mm, and they can be seen as coaxial. By using Equation (8), the coupling factor between tungsten coil and sensor coil can be calculated approximately to be 0.443. In Equation (8)*l* is the distance between the sensor and antenna, *r_1_* and *r_2_* are the radius of sensor coil and antenna separately:

(8) $$k(l)≅\frac{r_{1}^{2}\cdot r_{2}^{2}}{\sqrt{r_{1}\cdot r_{2}}{\left(\sqrt{l^{2}+r_{2}^{2}}\right)}^{3}}$$

**Figure 9.**
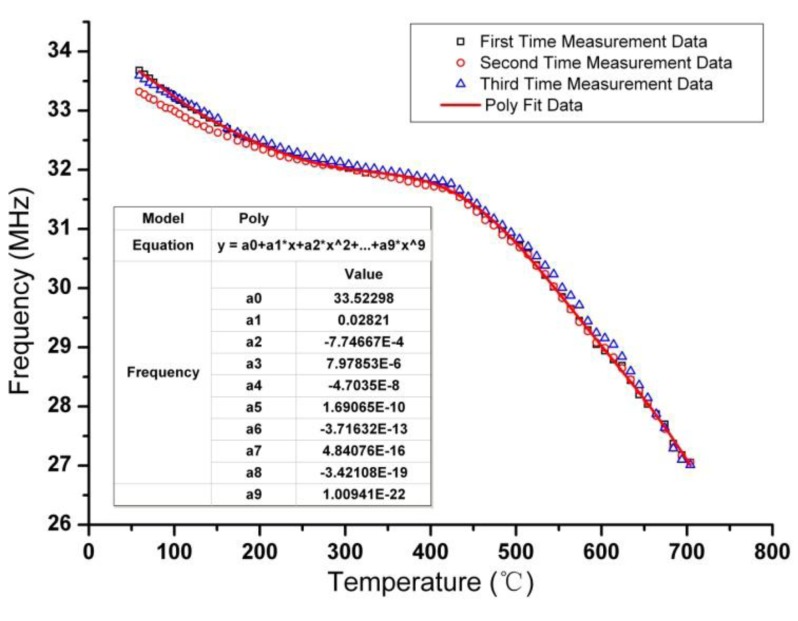
Measurement resonant frequency of the senor from room temperature to 700 °C.

The sensor and antenna are placed into the muffle furnace and the network analyzer is at room temperature environment. By leading two ends of the antenna out of furnace through a hole on the furnace door, and connecting the two ends to the network analyzer, the resonant frequency is obtained by retrieving the minimum impedance phase of the antenna. Measurements were repeated three times and the fitting data are illustrated in Figure 9, from which the decrease of resonant frequency with the increase of temperature and good repeatability of the sensor are shown. From the data of the three measurements, the repeatability error of the sensor is calculated to be 5.47%. The sensitivity of the sensor is about −5.75 KHz/°C before 430 °C, and −16.67 KHz/°C from 430 °C to 700 °C. The maximum data deviation occurs in the initial segment of temperature rise, it is due to the imprecise temperature control of muffle furnace in the initial segment.

**Figure 10.**
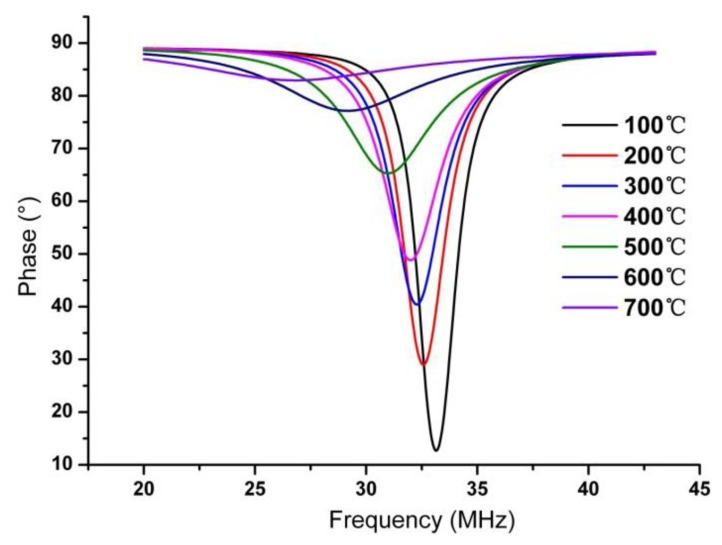
Measurement impedance phase *versus* frequency in different temperature.

As illustrated in Figure 9, the fitting curve can be expressed as:

(9) $$y=a_{0}+a_{1}x+a_{2}x^{2}+\cdots +a_{9}x^{9}$$

where *y* is resonant frequency of the sensor and unit MHz is used in this paper, *x* is temperature, *a_0_* to *a_9_* are multinomial coefficients and their values are also shown in Figure 9. As the increase of temperature, *Q* factor of the sensor decrease, which is reflected by the magnitude of phase difference. According to Equation (3), the decrease of *Q* factor is due to two major factors, the first is the resistivity increase of metallic silver, at the same time, the increscing permittivity of the ferroelectric and LTCC ceramic, which results in the increased capacitance *C_s_*, which also causes the decrease of *Q* factor. The measured phase curve *versus* frequency at different temperatures is illustrated in Figure 10.

**Figure 11.**
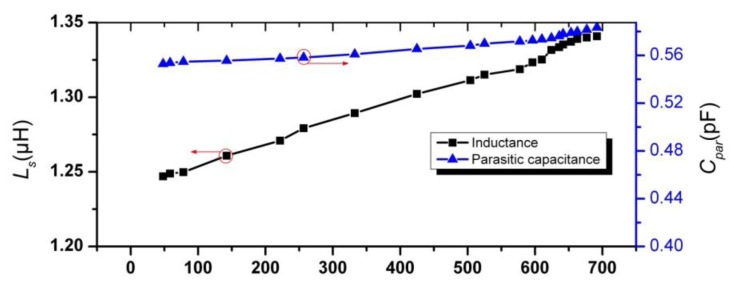
Measurement inductance and parasitic capacitance *versus* different temperature.

**Figure 12.**
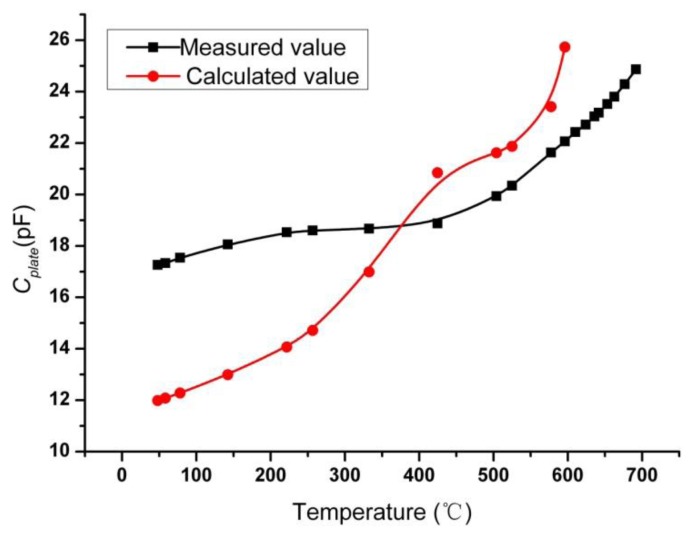
The capacitance of parallel capacitor *C_plate_ versus* different temperature.

By fabricating the coil with same dimensions as the sensor coil on the LTCC substrate, inductance and parasitic capacitance *versus* different temperature were measured and illustrated in Figure 11. It can be seen that the measured *L_s_* is smaller than the calculated value by Equation (6), which is mainly due to the shrinkage of dimensions after sintering and the influence of parasitic effects. By combining the measured resonant frequency, inductance and parasitic capacitance, the capacitance of parallel capacitor *versus* different temperature are deduced by Equation (2) and shown in Figure 12. Also, the calculated value of *C_plate_* by Equation (7) is illustrated in Figure 12. There is comparatively large deviation between the calculated value and measured value. The reasons for this deviation is due to cave-in of upper capacitor plate and the out of truth for the measurement values of permittivity of LTCC and ferroelectric ceramic may cause this great deviation between calculated value and measured value. The effects of ion-conductivity at elevated temperature may make the sensor perform worse than expected [18].

**Figure 13.**
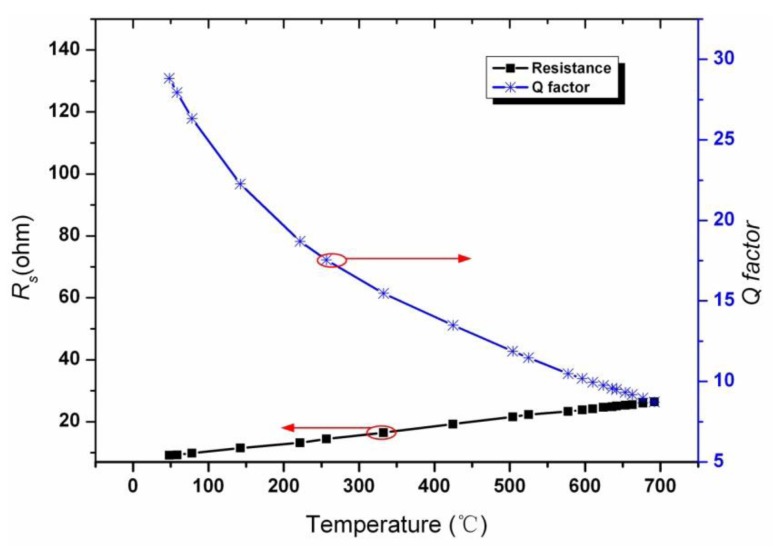
Measured resistance *R_s_* and *Q* factor *versus* different temperature.

The resistance of the coil *R_s_ versus* different temperatures is also measured by an Agilent E5061B Network Analyzer. By substituting the measured resistance, resonant frequency and inductance into Equation (3) in the paper, the *Q* factor *versus* temperature was obtained. The measured resistance and *Q* factor of the sensor are shown in Figure 13, it can be obtained that the *Q* factor of the sensor decreases from 28.8 to 7.83 when temperature increases from room temperature to 700 °C.

## 5. Conclusions and Future Works

This paper proposed a wireless passive temperature sensor based on a ferroelectric dielectric ceramic, which can be used for monitoring temperatures in harsh environments. Temperature sensitive high Curie ferroelectric dielectric ceramic is filled into the capacitor cavity and co-fired with a LTCC substrate. A fabrication process which is compatible with standard LTCC technology is described in detail in this paper. By photomicrography of the cross-section of the sensor sample, fullness of dielectric ceramic in LTCC cavity after co-firing with the LTCC substrate can be seen. The resonant frequency of the sensor is measured from room temperature to 700 °C three times. By analyzing the measurement data, considerable repeatability of the sensor can be seen. Sensitivity of the sensor is about −5.75 KHz/°C before 430 °C, and −16.67 KHz/°C from 430 °C to 700 °C. A nonlinear fitting is done, from which the measured data can be fitted by a ninth degree polynomial.

In future work, a ceramic dielectric material with higher Curie point and sensitivity should be used to make the sensor perform well, and the optimized dimension design of the sensor should also be investigated. At the same time, readout circuits and signal processing techniques with high resolution will be developed to increase the sensing range. At last, maximization of quality factor should be investigated to extend the measurement range.

## Figures and Tables

**Table 1.** Key design dimensions for the sensor.

**Symbol**	**Dimension Parameter**	**Value**
*w*	Width of the coil trace	0.3 mm
*s*	Coil trace spacing	0.57 mm
*n*	Number of turns of the coil	8
*r_s_*	Inner radius of the coil	5.45 mm
*a*	Length of the capacitor electrode	7 mm
*d*	Length of the square-shaped ferroelectric ceramic	5 mm
*t*	Thickness of the LTCC sheet	96.9 μm
*h*	Thickness of the ferroelectric ceramic	0.45 mm
